# Genomic epidemiology of dengue in Shantou, China, 2019

**DOI:** 10.3389/fpubh.2023.1035060

**Published:** 2023-07-13

**Authors:** Lina Tian, Chumin Liang, Xiaorong Huang, Zhe Liu, Juan Su, Chuan Guo, Guanghu Zhu, Jiufeng Sun

**Affiliations:** ^1^Guangdong Provincial Center for Disease Control and Prevention, Guangdong Provincial Institute of Public Health, Guangzhou, China; ^2^Guangdong Provincial Center for Disease Control and Prevention, Guangzhou, China; ^3^School of Mathematics and Computing Science, Guilin University of Electronic Technology, Guilin, China; ^4^School of Public Health, Southern Medical University, Guangzhou, China; ^5^Center for Disease Control and Prevention of Shantou City, Shantou, Guangdong, China

**Keywords:** dengue fever, dengue virus, epidemiology, genome, outbreak

## Abstract

**Objectives:**

Dengue has been endemic in Southeast Asian countries for decades. There are few reports tracing the dynamics of dengue in real time. In this study, we generated hundreds of pathogen genomes to understand the genomic epidemiology of an outbreak in a hyper-endemic area of dengue.

**Methods:**

We leveraged whole-genome short-read sequencing (PE150) to generate genomes of the dengue virus and investigated the genomic epidemiology of a dengue virus transmission in a mesoscale outbreak in Shantou, China, in 2019.

**Results:**

The outbreak was sustained from July to December 2019. The total accumulated number of laboratory-confirmed cases was 944. No gender bias or fatalities were recorded. Cambodia and Singapore were the main sources of imported dengue cases (74.07%, *n* = 20). A total of 284 dengue virus strains were isolated, including 259 DENV-1, 24 DENV-2, and 1 DENV-3 isolates. We generated the entire genome of 252 DENV isolates (229 DENV-1, 22 DENV-2, and 1 DENV-3), which represented 26.7% of the total cases. Combined epidemiological and phylogenetic analyses indicated multiple independent introductions. The internal transmission evaluations and transmission network reconstruction supported the inference of phylodynamic analysis, with high Bayes factor support in BSSVS analysis. Two expansion founders and transmission chains were detected in CCH and LG of Shantou.

**Conclusions:**

We observed the instant effects of genomic epidemiology in monitoring the dynamics of DENV and highlighted its prospects for real-time tracing of outbreaks of other novel agents in the future.

## Introduction

Dengue is a vector-borne disease caused by the dengue virus (DENV) and is mainly transmitted by *Aedes aegypti* and *Ae. albopictus* in tropical and subtropical countries. The manifestations of dengue illness are classified in the clinic as asymptomatic infection, dengue fever, dengue hemorrhagic fever (DHF), and dengue shock syndrome (DSS) according to the disease severity (1). The global incidence of dengue has increased by 30-fold over the past five decades (2). It has become one of the most serious global public health concerns, threatening ~2.5 billion individuals and infecting over 100 million people annually (3, 4). Although the first dengue vaccine CYD-TDV has been licensed (5), it is limited by prescreening for serostatus, and only seropositive individuals can receive the vaccination.

The causative agents of dengue include four distinct serotypes (DENV 1-4) according to their antigenicity (6). The cross-protection of antibodies among different serotypes only lasts for a few months (7), and secondary infection with divergent serotypes may lead to DHF, DSS, and even death (1). Therefore, timely detection and tracking of dynamic trends in dengue outbreaks will be the key to precise prevention and control. Laboratory techniques (including serological tests, nucleic acid-based real-time PCR, and partial gene sequencing) are commonly used for fast diagnosis or serotype determination but are incapable of discriminating particular genotypes or circulating variants. The current strategies of dengue prevention and control are still focused on vector control and case management. This is far from the goal of precise prevention and control of the infectious disease expected by the World Health Organization (WHO). Therefore, there is a great need to develop strategies to track the dynamics of highly transmissible diseases in real time.

Next-generation genome sequencing techniques, e.g., Illumina and Nanopore, coupled with empirical epidemiology facilitated fast-tracking dynamics of explosive outbreaks of highly pathogenic pathogens, including Middle East Respiratory Syndrome (MERS) in 2013 (8), Ebola fever in 2014 (9), ZIKV disease in 2016 (10–12), and the current global circulation of SARS-CoV-2 since 2019 (13). These techniques were also able to monitor any potential mutations that may convert the disease pathogenesis or alter the sensitivity to antibody-mediated immunity through vaccination or natural infection of target pathogens. An outstanding online platform, Nextstrain (www.nextstrain.org), was developed to allow the exploration of continually up-to-date datasets (14). Although Nextstrain provides a real-time snapshot of evolving pathogen populations through interactive data visualizations, local dynamic surveillance of pathogens still needs further evaluation.

Guangdong Province is part of the hyperepidemic dengue region in Southeast China. Although outbreaks of dengue are reported almost annually in Guangdong and neighboring Guangxi, Fujian, and Yunnan provinces, Guangdong still ranks at the top of most hyperepidemic dengue areas in China. More than 0.72 million dengue cases have been reported in this province, accounting for over 90% of the total archived dengue cases in China during the last four decades (15–17). However, dengue is an arbovirus triggered by imported cases each year. Sustained transmission of dengue to the next epidemic season is occasional and rare in Guangdong, China (15, 18, 19). Thus, early detection of imported cases and tracking dynamics of DENV is the key to containing the local transmission of dengue. In this study, we tracked a short-term, mesoscale dengue outbreak in Shantou (a prefecture-level city in Guangdong) in 2019 by using a real-time genome epidemiology approach. Beyond empirical strategies, real-time genomic epidemiology has proven to be the key to future precise prevention and control of highly transmissible diseases through transmission route tracking, including but not limited to dengue and the currently circulating coronavirus disease 2019 (COVID-19).

## Methods

### Ethics statement

All serum sampling protocols for dengue cases were approved by the Ethics Committee of the Guangdong Provincial Centers for Disease Control and Prevention (Guangdong CDC). The sampling procedures were performed in a local hospital in accordance with humanization regulations. Epidemiological, demographic, and clinical data of dengue cases were recorded by local hospitals in Shantou, Guangdong, China. The data were uploaded to the Notifiable Infectious Disease Report System (NIDRS) of China by local hospitals.

### Case definition

Dengue cases were defined according to the Chinese national criteria for dengue diagnosis (WS216-2008) and the 2009 World Health Organization (WHO) guidelines, which included suspected and confirmed cases (19, 20). A clinically suspected dengue case was defined as a patient who: (1) lived in or had a history of travel to a dengue-endemic area; (2) had a high fever of 39°C for 3 days or more, accompanied by two of the following criteria: nausea, vomiting, rash, severe headache, muscle and joint pain, or positive tourniquet test; and (3) had low or decreasing white cell counts and/or thrombocytopenia. A confirmed dengue case was a suspected case confirmed by a laboratory-positive diagnostic test, including the identification of DENV RNA (real-time RT–PCR or genome sequencing) or DENV NS1 antigen.

### Laboratory diagnosis, viral identification, and genome sequencing

Primary laboratory diagnosis of dengue cases was performed at Shantou Local Hospital. Patients were tested for the presence of IgM antibodies using commercial ELISA kits (PanBio, Windsor, Australia). Universal real-time reverse-transcriptase polymerase chain reaction (rRT-PCR) was used to initially detect DENV in acute-phase serum specimens. The serum samples that tested positive for viral RNA were transferred to the Guangdong CDC and used for virus isolation. The criteria for serum sample selection were based on the guidelines for dengue prevention and control in Guangdong, China. Samples with low serum volume and low virus load were excluded. The selected samples were centrifuged at 250 × g for 10 min. The suspension was filtered through a 0.22 μm filter, collected, and used for subsequent virus culture. Next, Vero E6 cells were inoculated with 100 μl of treated patient samples. The cytopathic effect was observed daily until the seventh day. The cell culture supernatants were harvested and stored at −80°C until used for genome sequencing (19). All the above laboratory practices related to clinical samples, cultures, and isolates were carried out in the Biosafety Level 2 (BSL-2) laboratory according to the guidelines issued by China CDC (WS216-2008). The protocols, emergency plan, and precaution strategies of the above practices were evaluated and approved by the biosafety committee of Guangdong CDC.

The total RNA of isolates was extracted by TaKaRa MiniBEST Viral RNA/DNA Extraction Kit Ver.5.0. The purity and concentration of the total RNA were detected by a Thermo NanoDrop One Qubit 3.0 (Thermo Fisher Scientific, MA, USA). The qualified RNA samples were randomly interrupted at a high temperature to produce RNA fragments of the required length for collection, and double-stranded cDNA was obtained by reverse transcription and second-strand synthesis. The library was established based on the NEBNext^®^ Ultra™ II DNA Library Prep Kit for Illumina^®^ (New England Biolabs, USA) standard process. PE150 sequencing was performed on the constructed library using the Illumina Nova 6000 platform.

Raw sequencing reads were checked through a quality control pipeline using SOAPnuke v2.0.5. Low-quality reads with a score > 20, N proportion <3% and A proportion > 40% were filtered. Adaptors were removed simultaneously. Repeated reads were removed through readfq v5.2. Clean reads were aligned with the reference genome (NC_001474.2 Dengue virus) by BWA. Matched reads were assembled by Megahit v1.2.9.

### Phylogenetic analysis

Assembled genomes were aligned with reference genomes of DENV1-4. Serotypes were determined according to similarity with the reference genomes (>95%) (21). Then, assembled genomes were aligned separately using the MEGA version 6 (22). The similarity of the assembled sequences was searched in the GenBank database (www.blast.ncbi.nlm.nih.gov/Blast.cgi) to select the reference genome for further phylogeny and phylodynamic analysis. The closest dengue genome sequences were initially selected by the neighbor-joining approach using MEGA, and the closely related isolates in the same or neighboring clusters were used for further analysis.

Finally, 1,439 DENV 1, 1,060 DENV 2, and 797 DENV 3 reference genomes were selected for accurate phylogenetic reconstruction to determine the genotypes of each isolate. The general time reversible (GTR) nucleotide substitution model with a proportion of invariant sites was identified again as the best-fitting model for ML inference by jModelTest v.1.6 (23). Maximum likelihood (ML) phylogenetic trees were inferred with Mega 6.06 using the general time reversible model and a gamma distribution (G+I, 4 nucleotide substitution model) with 1,000 bootstraps.

### Phylodynamics of DENV 1

The initial laboratory test showed that DENV 1 was the major outbreak serotype, with few DENV 2 and DENV 3. Therefore, DENV 1 was selected as an example to draw the phylodynamic trends of this outbreak. The temporal signal analysis of the correlation between the root-to-tip genetic divergence of DENV 1 and the date of sampling was conducted in TempEst (24). The correlation between the sampling date of each sequence and the genetic distance of that sequence from the root of a maximum likelihood phylogenetic tree for DENV 1 fell on the regression line. Recombination assessed using GARD from the Data Monkey software suite did not indicate apparent recombination in the DENV 1 dataset (25).

Bayesian phylogenies were estimated in BEAST v.1.10.4 using relaxed uncorrelated molecular clock (UCLN) models under the GTR nucleotide substitution model (26, 27). Three independent Markov chain Monte Carlo (MCMC) runs of 100 million steps were computed with ESS > 200, and a 10% burn-in was discarded from each run. The convergence and behavior of the MCMC were inspected using Tracer v1.6 (http://beast.bio.ed.ac.uk/Tracer). The summary phylogenies were visualized in FigTree v.1.4.2 (http://tree.bio.ed.ac.uk/software).

To identify well-supported transmission rates between locations in standard discrete phylogeographic reconstructions, we calculated the subset of location exchange rates that dominated the diffusion process using the Bayesian stochastic search variable selection (BSSVS) procedure. The Bayes factor (BF) from the BSSVS analysis was determined using SpreaD3, a tool for analyzing and visualizing discrete and continuous trait evolutionary histories associated with phylogenies (28).

### Transmission network reconstruction

The dengue transmission network was reconstructed by using Population Analysis with Reticulate Trees (POPART), a software package for population genetics analysis using haplotype networks (www.popart.otago.ac.nz). A TCS network was constructed with default parameters (29, 30). All 229 DENV 1 genomes were aligned to the consensus and trimmed to the same length, yielding aligned sequences of 10,605 base pairs (bp). To be as conservative as possible in mutation calling, gaps and non-identifiable positions were assumed to have major alleles. Genomic positions without variation were removed.

## Results

### Epidemiology findings

**Figure 1:**
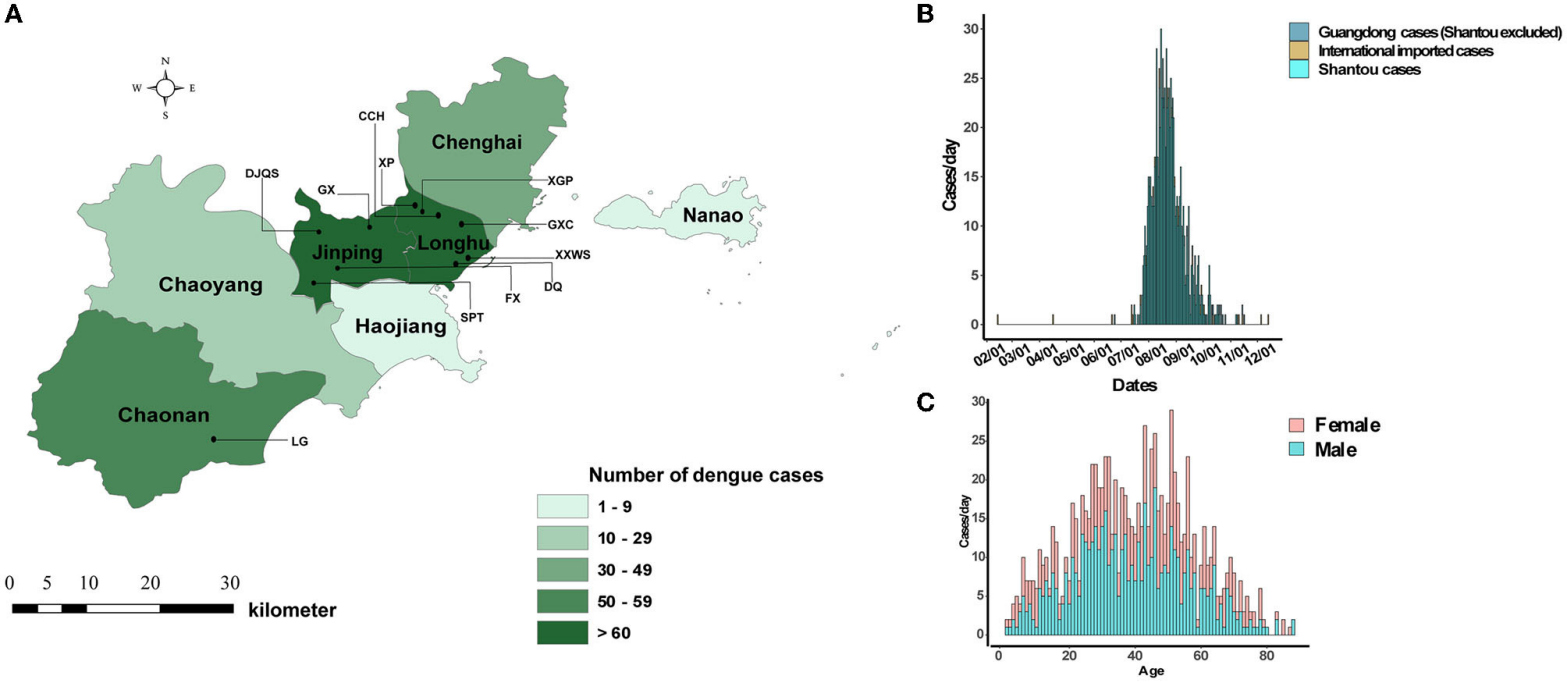
Geographical, epidemiological, and gender distribution of dengue cases in Shantou, China, 2019. **(A)** Eight districts of Shantou and live transmission communities of dengue. **(B)** Time series distribution of imported and local dengue cases. **(C)** Gender distribution with age.

An unexpected dengue outbreak occurred in Shantou, initially starting in July and continuing until December 2019. The accumulated 944 cases were concentrated in an area of 2,064 km^2^, and seven districts were included (Figure 1A). The daily time course of the recorded dengue cases showed that the explosive outbreak was detected in July and reached a peak level in the middle of August (Figure 1B). Of the total 944 cases, 539 (57.09%) and 405 (42.91%) occurred in men and women, respectively. The median age of the patients was 40 years old (range 1–88). The most affected age group was 20–60 years old (Figure 1C). No fatalities were recorded. Unemployed individuals (324, 34.32%), workers (124, 13.14%), students (98, 10.38%), and retailers (80, 8.47%) were the most affected populations. Most patients were from CCH (263, 27.68%), XGP (166, 17.58%), DJQS (115, 12.18%), and FX (76, 8.05%), which are located in the Longhu and Jinpin districts of Shantou. Additionally, 27 were foreign travelers who came from Cambodia and Singapore (74.07%, *n* = 20), and 17 were domestic travelers who came from other provinces in China (Table 1).

**Table 1:** Summary of imported DENV infection cases in Shantou, 2019.

	**Guangzhou**	**Fujian**	**Xiamen**	**Yunnan**	**Dongguan**	**Foshan**	**Chaozhou**	**Shantou**	**Total (27)**
Thailand	0	0	0	0	0	0	0	4	4
Cambodia	0	0	0	0	0	0	0	17	17
Malaysia	0	0	0	0	0	0	0	1	1
Indonesia	0	0	0	0	0	0	0	1	1
Philippines	0	0	0	0	0	0	0	4	4
Total (17)	6	1	1	1	4	1	2	1	

All 944 dengue cases were laboratory-confirmed cases verified by real-time RT–PCR (871, 72, and 1 for DENV 1, 2, and 3, respectively). A total of 398 serum samples from Shantou were submitted to the Guangdong CDC. A total of 284 DENV isolates were successfully obtained, including 259 DENV 1, 24 DENV 2, and 1 DENV 3 isolates.

### Infection status of viral RNA in serum

**Figure 2:**
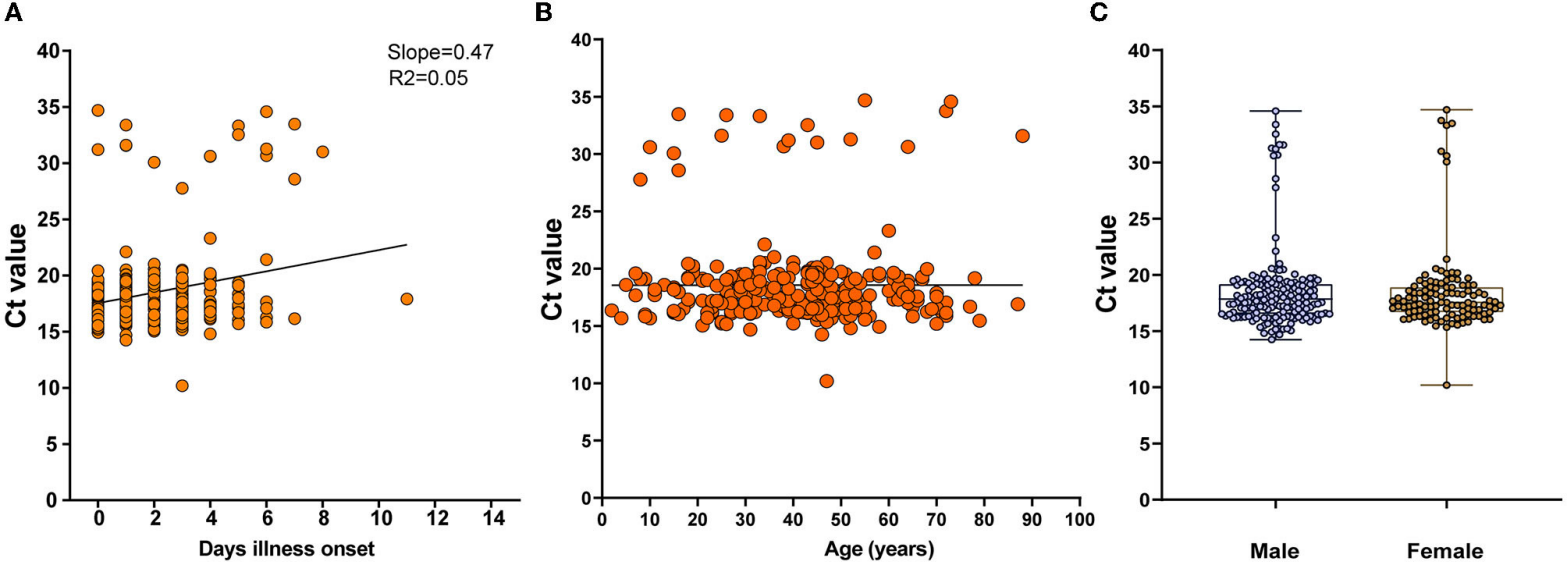
Dynamic shedding of viral RNA in serum. **(A)** The time cost for dengue patients to clear viral RNA in days since illness onset, which is shown as increasing cycle threshold (Ct) values tested by rRT-PCR. **(B, C)** Associations between Cts and age or gender.

An understanding of the dynamics of the stages of DENV infection is needed to inform diagnostic testing and prevention interventions. We determined the time cost for dengue patients to clear viral RNA through observation of the accelerated failure time, which showed increasing cycle threshold (Ct) values tested by rRT-PCR (slope = 0.47, R^2^ = 0.05). The viremia period was mainly sustained in the first week after symptom onset (mean Ct and SD were 18.56, and 3.767, respectively) (Figure 2A). No associations were detected between Cts and age or sex (Figures 2B, C).

### Phylogenetic analysis

All 284 DENV isolates were sent for genome sequencing, resulting in 252 whole sequences (229 DENV 1, 22 DENV 2, and 1 DENV 3), which represented over 26.7 % of the total outbreak cases. Three DENV serotypes were identified including DENV 1 to 3. Phylogenetic analysis for each DENV serotype showed that multiple resources likely contributed to this outbreak. For instance, 229 DENV 1 isolates were assigned to a single cluster in genotype I, which was closely related to genomes from Cambodia and Singapore (Supplementary Figure 1). Twenty-two DENV 2 isolates were assigned to a single cluster of the Asia I genotype, and the closest genomes were from Singapore in 2004–2006 (Supplementary Figure 2). A unique DENV 3 outbreak strain was isolated from imported cases that traveled in Malaysia, which belonged to genotype I of DENV 3 (Supplementary Figure 3).

### Phylodynamics of DENV 1

**Figure 3:**
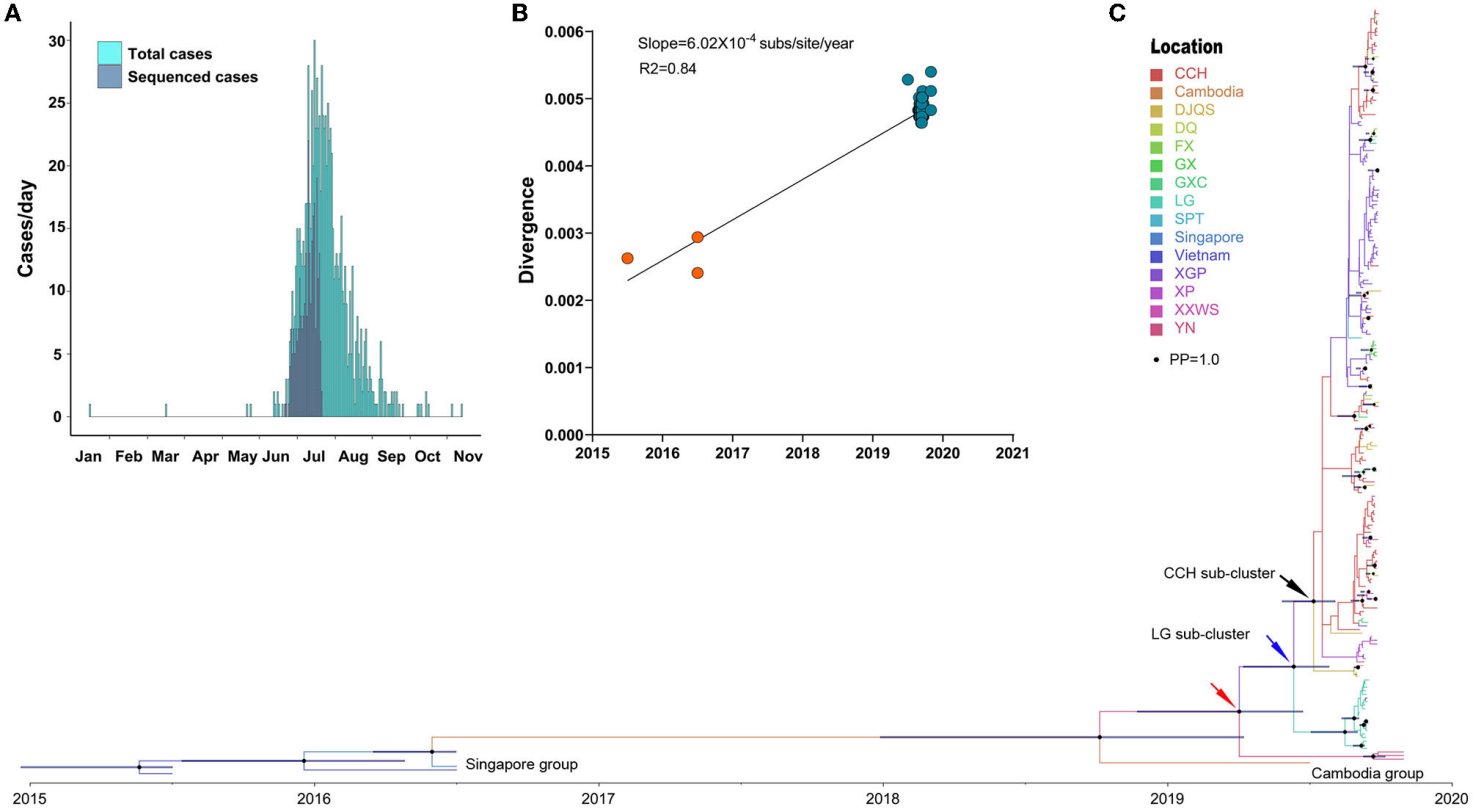
Phylogenetic characteristics of DENV 1. **(A)** Distribution of the generated DENV 1 genome during the outbreak. **(B)** Genomic distance divergence through root-to-tip assay. **(C)** Bayesian evolution of DENV 1. Source countries/regions of each strain and different genotypes of DENV 1 are presented as different colors. Dots are internal branch points with posterior probability ranging from 0.9 to 1.0 with dot size. The red arrow indicates the common ancestor cluster of this outbreak. The blue and black arrow indicates two separate subclusters DENV 1 in LG of Chaonan and CCH of Jinpin district, respectively.

Phylodynamic analysis of 229 DENV 1 genomes represented 26.29% (259/871) of DENV 1-infected cases (Figure 3A), which were mainly distributed from July to August 2019. The temporal signal analysis of the root-to-tip genetic divergence indicated a linear correlation between the sampling date of each sequence and the genetic distance of that sequence from the root of a maximum likelihood phylogenetic tree of DENV 1 (slope = 6.02 × 10^−4^, R^2^ = 0.84) (Figure 3B). The estimated evolutionary rate of DENV 1 under the selected evolutionary model was 1.96 × 10^−3^ (95% HPD: 9.91 × 10^−4^ – 3.12 × 10^−3^) s/s/y (Figure 3C). The corresponding estimated time of the most recent common ancestors (tMRCAs) was 2015 (95% HPD: 2,014.97–2,015.50).

The time-scaled phylodynamic analysis identified a single cluster of DENV 1 during the outbreak (posterior probability, PP = 1.0) and revealed the extensive transmission of DENV 1 from LG of Chaonan to CCH of Jinpin, and to other districts (Figure 3C). The estimated tMRCA of a single outbreak cluster was March 2019 (95% HPD: March 2019–April 2020) (Figure 3C, red arrow). The most common ancestor of this cluster was likely from Cambodia in 2019, which was also exported to Yunnan, China, in July of the same year. Moreover, two separate subclusters with high posterior probability support (PP = 0.98 and 0.96) indicated two sequential independent outbreaks of DENV 1 in LG of Chaonan (Figure 3C, blue arrow) and CCH of Jinpin district (Figure 3C, black arrow), respectively. The estimate of the tMRCA of the CCH cluster was June 2019 (95% HPD: June–November 2019). The tMRCA of the LG cluster was July 2019 (95% HPD: June–November 2019). The most common ancestor of these two clusters was likely from Cambodia in 2019. In particular, within the CCH subcluster, there was another potential XGP subcluster based on the geographical distribution of cases. However, it was not supported by the posterior probability test (PP = 0.01).

**Figure 4:**
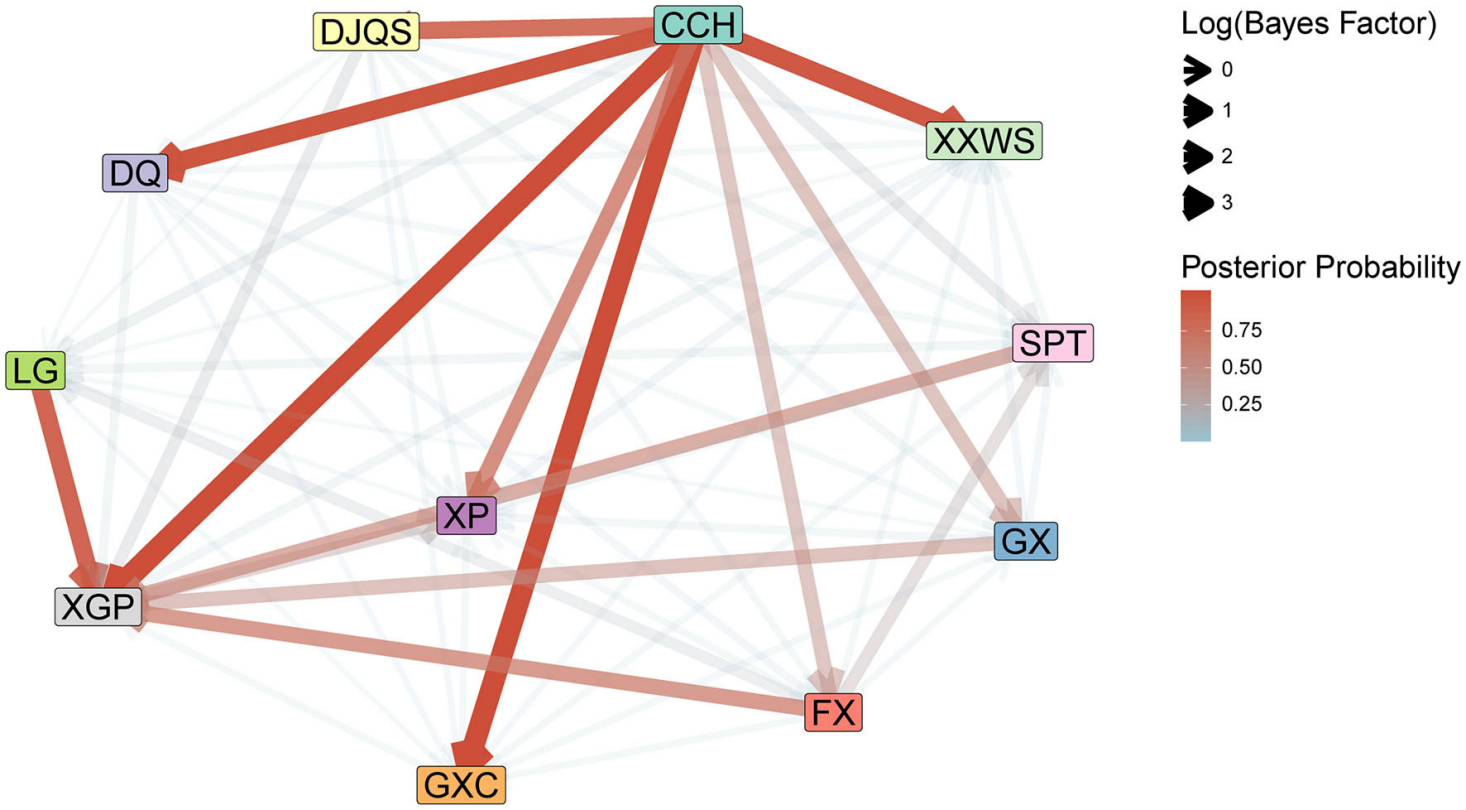
Internal transmission evaluation of DENV 1 with BSSVS analysis in 11 communities of Shantou, 2019. The Bayes factor and posterior probability were summarized to indicate the direction of virus transmission and branch support.

The internal transmission evaluations of the outbreak support the inference of the phylodynamic analysis (Figure 4). The Bayes factors from the BSSVS analysis indicated two expansion founders, CCH and LG, with high Bayes factor support. CCH was the most frequent export location to DJQS (BF = 29.01), DQ (BF = 152.56), XGP (BF = 4,657.79), GXC (BF = 1,771.83), and XXWS (BF = 115.39) of the Jinpin and Longhu districts, while another founder, LG of Chaonan, only exported to GXC of the Jinpin district (BF = 46.19). In addition, the Bayes factors of BSSVS analysis showed a rather high support of transmission (BF = 4,657.79) from CCH to XGP, which indicated a close transmission from each other.

### Transmission network reconstruction of DENV 1

**Figure 5:**
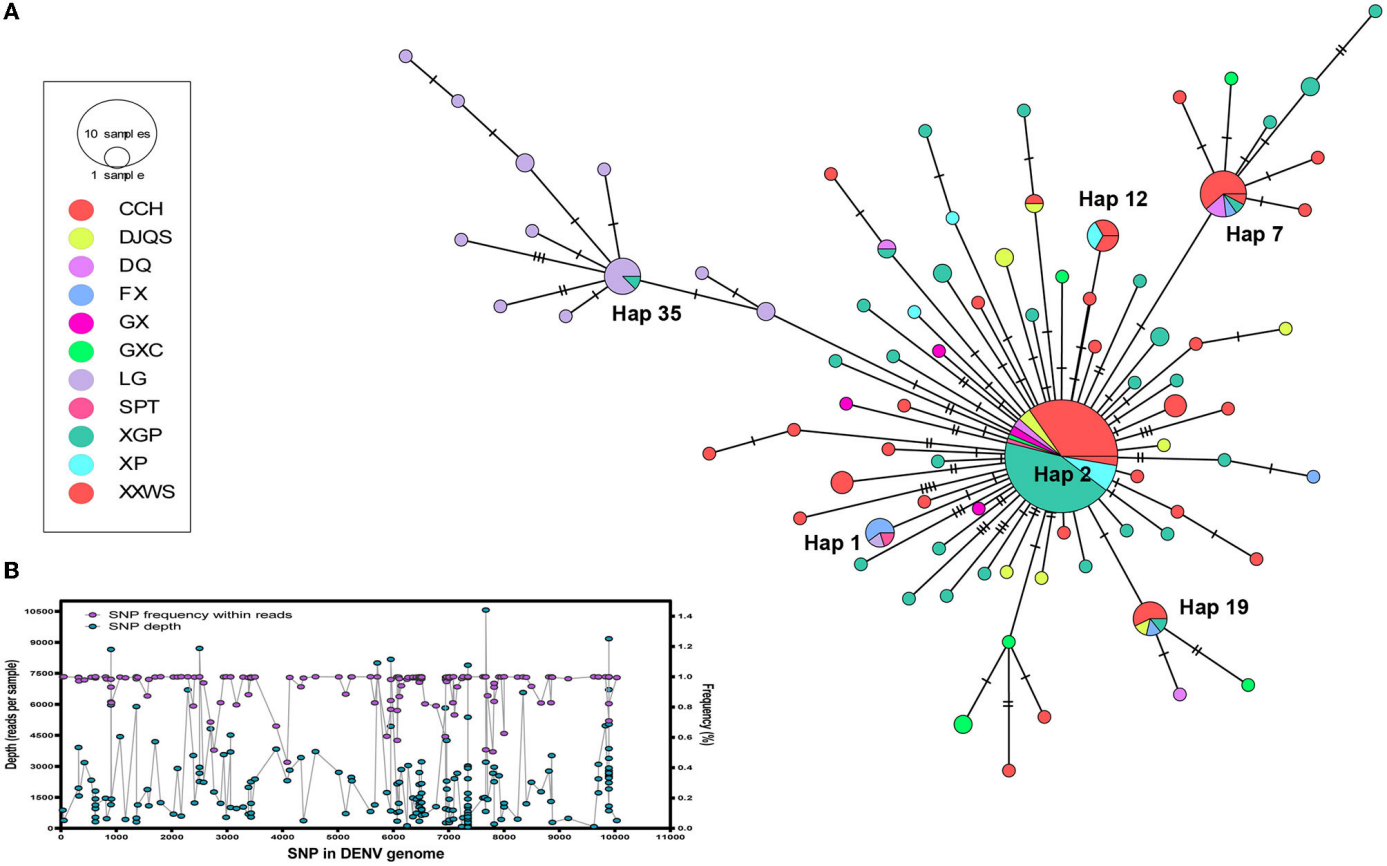
Transmission network reconstruction of DENV 1. **(A)** Transmission network of dengue in 11 communities of Shantou, 2019. The circle size indicates the isolates from each haplotype. Colors represent the communities. **(B)** The depth and frequency of each SNV in the DENV 1 genome showed the quality of each SNP. Maximum likelihood (ML) tree reconstruction using MEGA with general time reversible (GTR) nucleotide substitution model with a proportion of invariant sites, 1,000 bootstraps.

The DENV 1 genome was assigned to 82 haplotype (Hap) viruses based on 111 single-nucleotide variants (SNVs) in the POPART assay (Figure 5A). The depth and frequency of each SNV indicated a high quality of each SNP, which represents reliable mutation rather than sequencing error (Figure 5B). The TCS network showed a dynamic expansion of the present dengue outbreak. The most expanded clonal virus was Hap 2 (*n* = 78 isolates), followed by Hap 7 (*n* = 13), Hap 35 (*n* = 8), Hap 19 (*n* = 7), Hap 12 (*n* = 6), and Hap 1 (*n* = 5). The remaining Haps contained only single or double isolates (*n* = 167). Hap 2 was one initial outbreak founder and expanded to nine out of 11 districts of Shantou City, while the second initial founder, Hap 35, only circulated in LG of Chaonan and expanded to GXC of Jinpin district in Shantou City.

## Discussion

Dengue is an imported arboviral disease in Guangdong, China (15). Thus, an early diagnosis of imported cases and tracking dynamics of DENV is key to containing onward transmission of dengue (18). Archived molecular epidemiology studies focused on tracing the source of dengue outbreaks mainly employed the E gene, an envelope protein-coding gene on the hydroxyl terminus of the DENV genome (31–35). The divergence of the E gene within four dengue serotypes allowed us to discriminate different DENV genotypes in each outbreak and facilitated tracing the source of imported DENV. We previously reported the molecular evolutionary history of DENV in the third-largest outbreak of dengue in 2014 (36). In addition, we inferred double outbreak sources in Chaozhou and Shantou cities simultaneously through phylodynamic analysis of DENV 2 in 2015 (20). However, the limited phylogenetic information in these studies that employed the E gene of DENV only facilitates genotyping of outbreak isolates rather than tracing the transmission routes of DENV. The genomic epidemiology of dengue employs the whole genome of DENV, ~11 kb vs. ~1.5 kb RNA in the E gene. The accumulated mutations in the DENV genome would be favorable for both genotyping and inference of transmission routes within a single outbreak.

In this study, we first described the epidemiological findings of the dengue outbreak in Shantou in 2019 and found that the viremia period mainly lasted for approximately 7 days after symptom onset (mean Ct and SD, 18.56 and 3.767), which is consistent with the time of sustained clinical symptoms (37). Next, we found multiple serotype transmissions of dengue in this short-term outbreak, which highlights that fast molecular genotyping, will help epidemiological surveys of important resources. Indeed, we detected multiple serotype outbreaks of dengue in Guangdong in 2014 (36) and 2015 (20), which may infer the sustained circulation of four serotypes of dengue in exportation countries, e.g., Southeast Asia. The extended time-scaled phylodynamic analyses in this study focused on DENV 1 genomes, which represented over a quarter of DENV 1-infected cases, revealing multiple internal transmissions within different districts. However, few accumulated mutations in the DENV E gene in those studies make it difficult to infer any internal transmission routes within a single outbreak; thus, most dengue outbreak reports only focus on genotyping of particular DENV genotypes (38–40). Recently, a number of studies have focused on dengue outbreak investigations, and DENV genomic sequencing was recorded in Hainan and Xishuangbannan of China, as well as in Senegal (33, 41, 42). Whole genomes of partial outbreak isolates were sequenced, and the phylogeny tree was built to trace the origin of transmission. However, no DENV transmission dynamic inferences were conducted in those studies, which limits the usage of whole genome data of DENV. In 2022, Su et al. (43) developed a multiplex PCR strategy to obtain the whole genome from all four serotypes of DENV and evaluated it in clinical samples (threshold cycle range, 23.91–35.11; coverage, 97.34–99.52%; depth, 20). This makes it possible to sequence the whole genome of DENV in low-equipped laboratories. In the same year, Li et al. (44) reported a unified global genotyping framework of DENV-1 incorporating 5,003 DENV-1 strains from 78 epidemic countries/areas from 1944 to 2018, which laid a foundation and unveiled the urgency for establishing a stratified coordinated surveillance platform that could be utilized to quantitatively assess DENV-1 epidemics although it was constructed based on the E gene only. In this study, genome sequencing of DENV 1 yielded sufficient SNPs (*n* = 111), which were used not only for transmission dynamic inference with BSSVS analysis but also for haplotype transmission network reconstruction. The Bayes factors of BSSVS analysis data indicated two expansion founders, CCH and LG, with high Bayes factor support. Although the haplotype network only showed the enrichment of a particular virus isolation based on SNP variants, a TCS network indicated the same founders as BSSVS inference, CCH, and LG, which was consistent with the phylodynamic data. Our current investigation leveraging evolutionary analyses of Whole Genomes generated during the study provided novel insights into the disease transmission dynamics and expansion founders that were not detected previously with smaller datasets (38–40) and single genes (like E gene) in DENV (15).

Compared to multi-locus phylogenetic studies, which are normally used for large-size, complex-genome pathogens, e.g., bacteria or fungi, viruses have a rather small size and simple genome structure, which allows virus genomic epidemiology studies to be used for the fast-tracking of the dynamics of explosive outbreaks of arboviruses, respiratory viruses, and enteroviruses (45–48). A global outbreak of ZIKV occurred in 2016, and a number of genomic epidemiology studies addressed regional and global transmissions of this virus and determined potential mutations in the genome that may convert the pathogenesis *in vivo* (10, 49). Highly pathogenic avian influenza H7N9 was initially detected in Shanghai, China, and recurred in other regions of China from 2013 to 2017 (50). Genome-based epidemiology studies have drawn almost real-time transmission routes of H7N9 in China, which serve for precise prevention and control of this virus in China and other countries suffering from H7N9. Currently, genomic epidemiology is used for real-time tracing of the transmission of SARS-CoV-2 from the alpha to omicron variants (13). In addition, it could also detect any potential mutations that may convert the pathogenesis or cause immune evasion of a vaccine. Therefore, a genomic epidemiology approach will significantly increase the capacity to find novel infectious pathogens, as well as trace any outbreaks in real time. However, the limitations of this approach were also noticeable for most public health organizations, e.g., highly equipped laboratories, excellent trained technicians, and cross networks of laboratories. Fortunately, next-generation genome sequencing technology has become increasingly easily achieved, e.g., Nanopore (51), which allows it to be used in fieldwork with low demand for both laboratories and technicians. Our study adds to this literature by demonstrating the benefits of genome epidemiology toward disease monitoring in general and tracing the transmissions in moderate outbreaks of DENV in particular.

In conclusion, we presented the epidemiological and genomic phylogenetic characteristics of DENV outbreaks in Shantou, Guangdong, China, in 2019. We found that multiple serotypes of DENV contributed to this outbreak. The phylodynamic and network reconstruction indicated accurate local transmission of DENV, which highlights the extreme importance of real-time genome tracing studies during the prevention and control of any travel-associated infectious diseases, e.g., dengue, as well as for other explosive infectious disease agents. The significance of this study lies in the fact that it not only identified the genotypes of outbreak isolates but also determined the dynamic transmission route and transmission network, all of which will greatly facilitate the tracing of the sources of the outbreak and design timely mitigation strategies. It can serve as a guide for both epidemiological surveys and monitoring novel pathogens that may aggressively evolve in human infections in the future (52).

## Data availability statement

The datasets presented in this study can be found in online repositories. The names of the repository/repositories and accession number(s) can be found below: https://www.ncbi.nlm.nih.gov/bioproject/ and PRJNA974178.

## Ethics statement

The studies involving human participants were reviewed and approved by the Ethics Committee of Guangdong Provincial Center for Disease Control and Prevention. Written informed consent to participate in this study was provided by the participants' legal guardian/next of kin.

## Author contributions

JSun: conception, design, obtained funding, supervision, and had full access to all of the data in the study and takes responsibility for the integrity of the data and the accuracy of the data analysis. JSun and LT: statistical analysis. All authors: acquisition or interpretation of data, critical revision of the manuscript for important intellectual content, and final approval of the version to be published.

## Supplementary material

Supplementary Figure 1 The phylogenetic tree of DENV 1 was constructed by maximum likelihood (ML) tree reconstruction using MEGA with a general time reversible (GTR) nucleotide substitution model with a proportion of invariant sites, and 1,000 bootstraps. Genotypes were marked with an arrow, and the outbreak isolates were labeled with blue dots.

Supplementary Figure 2 The phylogenetic tree of DENV 2 was constructed by maximum likelihood (ML) tree reconstruction using MEGA with a general time reversible (GTR) nucleotide substitution model with a proportion of invariant sites, and 1,000 bootstraps. Genotypes were marked with an arrow, and the outbreak isolates were labeled with blue dots.

Supplementary Figure 3 The phylogenetic tree of DENV 3 was constructed by maximum likelihood (ML) tree reconstruction using MEGA with a general time reversible (GTR) nucleotide substitution model with a proportion of invariant sites, and 1,000 bootstraps. Genotypes were marked with an arrow, and the outbreak isolate was labeled with blue dots.
