# Supplementary figures and images for: Genomic epidemiology of dengue in Shantou, China, 2019
Source: Front Public Health. 2023 Jul 13;11:1035060. doi: 10.3389/fpubh.2023.1035060 (PMC10374217; doi:10.3389/fpubh.2023.1035060)

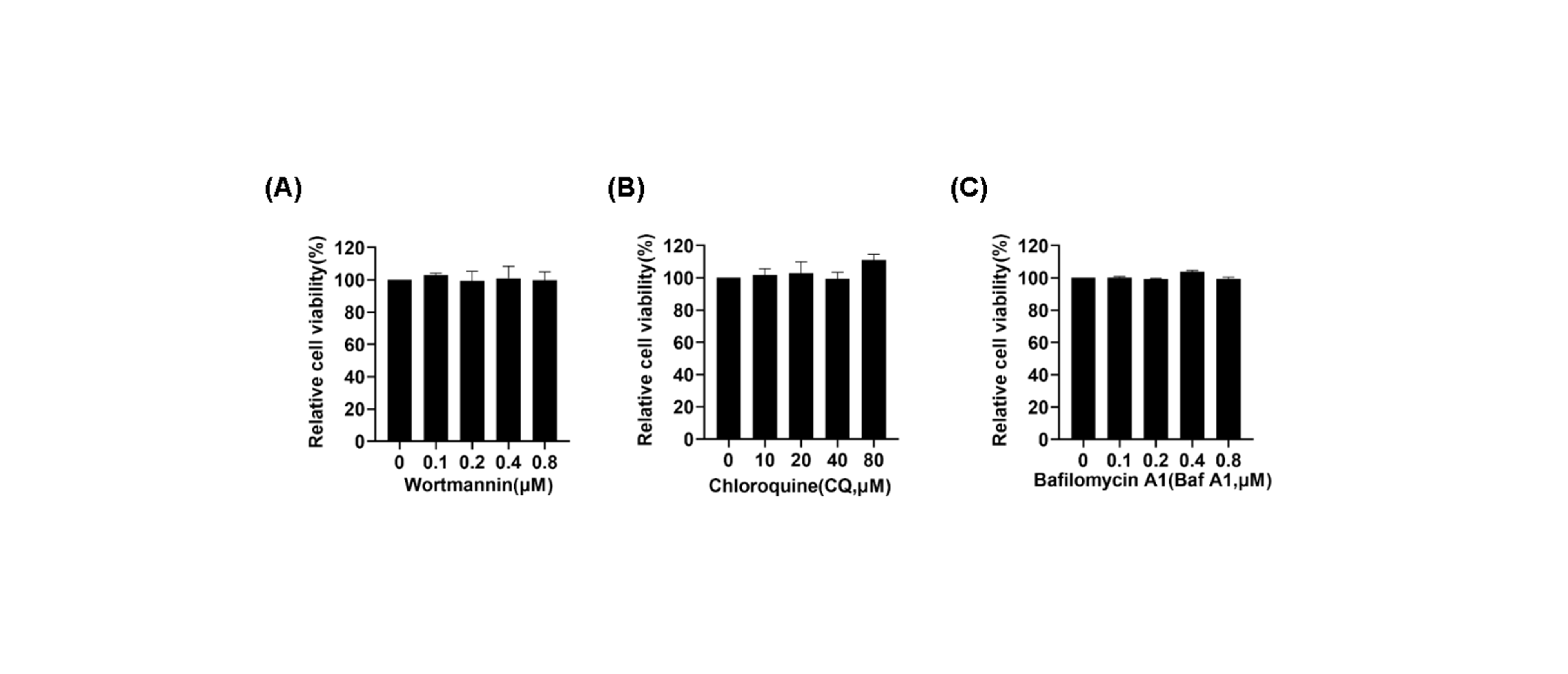

Supplement: Supplementary Figure 1 — The phylogenetic tree of DENV 1 was constructed by maximum likelihood (ML) tree reconstruction using MEGA with a general time reversible (GTR) nucleotide substitution model with a proportion of invariant sites, and 1,000 bootstraps. Genotypes were marked with an arrow, and the outbreak isolates were labeled with blue dots. [file Image_1.TIF]

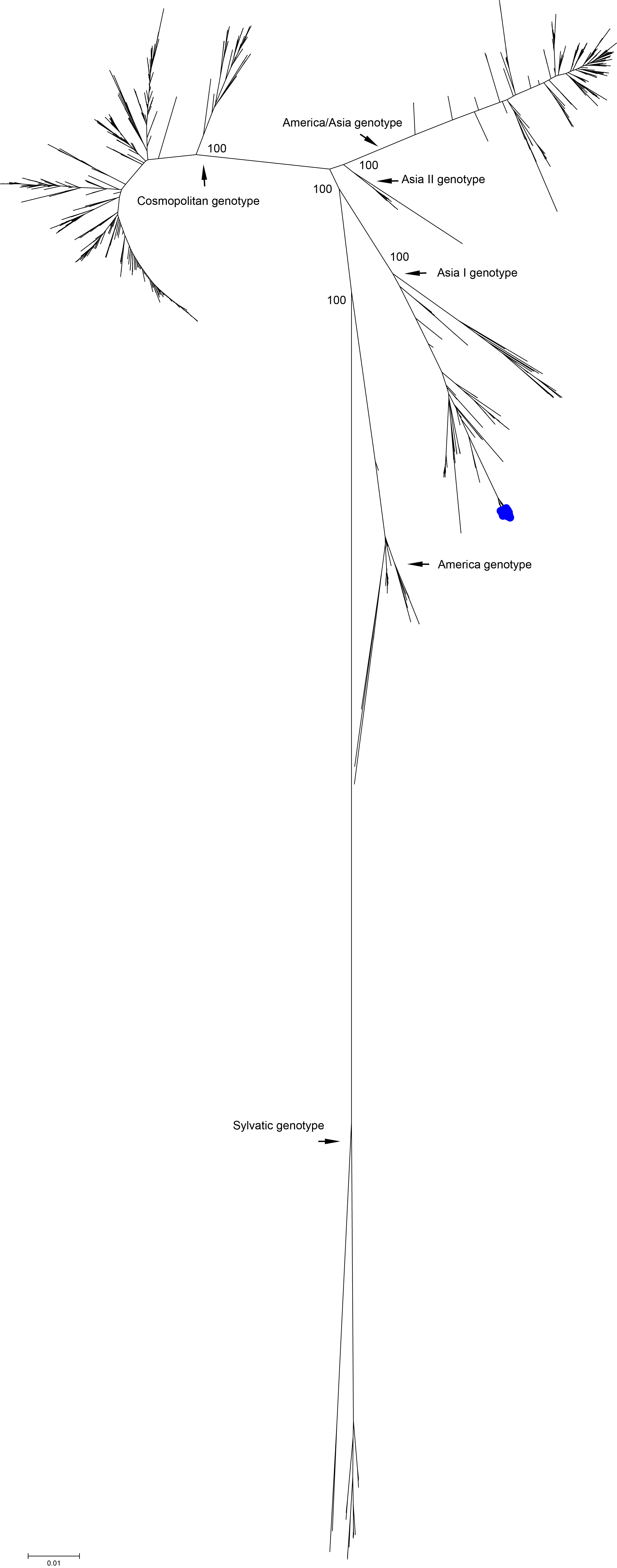

Supplement: Supplementary Figure 2 — The phylogenetic tree of DENV 2 was constructed by maximum likelihood (ML) tree reconstruction using MEGA with a general time reversible (GTR) nucleotide substitution model with a proportion of invariant sites, and 1,000 bootstraps. Genotypes were marked with an arrow, and the outbreak isolates were labeled with blue dots. [file Image_2.TIF]

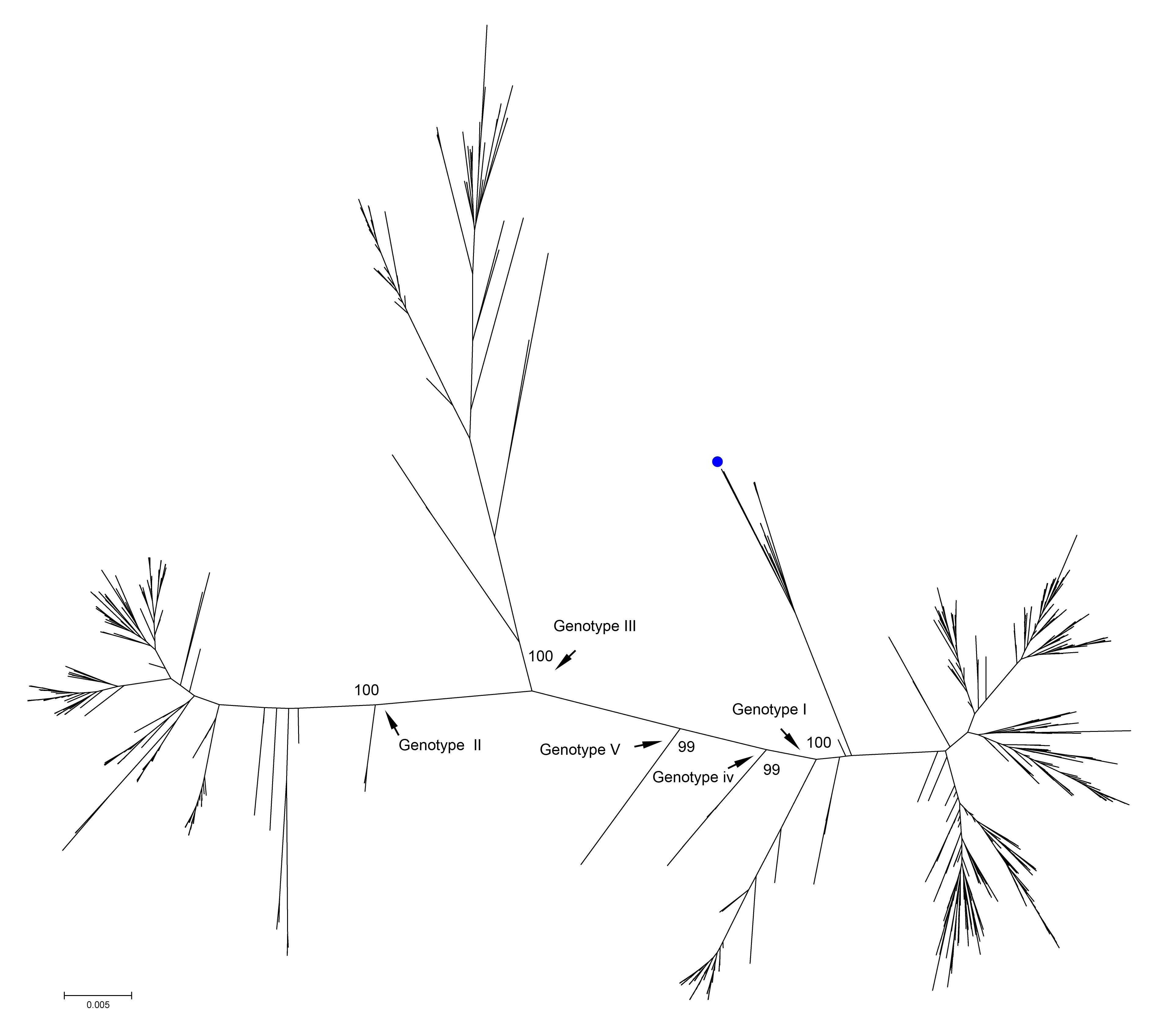

Supplement: Supplementary Figure 3 — The phylogenetic tree of DENV 3 was constructed by maximum likelihood (ML) tree reconstruction using MEGA with a general time reversible (GTR) nucleotide substitution model with a proportion of invariant sites, and 1,000 bootstraps. Genotypes were marked with an arrow, and the outbreak isolate was labeled with blue dots. [file Image_3.TIF]
